# *Taenia solium* taeniosis and cysticercosis literature in Tanzania provides research evidence justification for control: A systematic scoping review

**DOI:** 10.1371/journal.pone.0217420

**Published:** 2019-06-05

**Authors:** Helena Aminiel Ngowi, Andrea Sylvia Winkler, Uffe Christian Braae, Robinson Hammerthon Mdegela, Ernatus Martin Mkupasi, Mwemezi Lutakyawa Kabululu, Faustin Peter Lekule, Maria Vang Johansen

**Affiliations:** 1 Department of Veterinary Medicine and Public Health, Sokoine University of Agriculture, Morogoro, Tanzania; 2 Center for Global Health, Department of Neurology, Technical University of Munich, Munich, Germany; 3 Centre for Global Health, Department of Community Medicine and Global Health, Institute of Health and Society, University of Oslo, Oslo, Norway; 4 One Health Center for Zoonoses and Tropical Veterinary Medicine, Ross University School of Veterinary Medicine, Basseterre, Saint Kitts and Nevis; 5 Department of Infectious Disease Epidemiology and Prevention, Statens Serum Institut, Copenhagen, Denmark; 6 Tanzania Livestock Research Institute (TALIRI)—Uyole, Mbeya, Tanzania; 7 Department of Animal Science and Production, Sokoine University of Agriculture, Morogoro, Tanzania; 8 Department of Veterinary and Animal Sciences, Faculty of Health and Medical Sciences, University of Copenhagen, Copenhagen, Denmark; Sciensano, BELGIUM

## Abstract

**Background:**

Despite *Taenia solium* taeniosis/cysticercosis (TSTC) having been put high on the global agenda of neglected tropical diseases (NTDs), which over the last years has received a lot of attention, there has been no control programmes in place in sub-Saharan Africa, a highly endemic region. This could be attributed to lack of awareness of many stakeholders on the burden and impact of *T*. *solium*. This information is essential in guiding TSTC policies, practices and research agendas as well as encouraging cross-sectoral collaboration in the control of this important zoonotic parasite using a One Health approach. National elimination of the parasite is the foundation for global eradication. This will require that substantial country-level information is provided to all key stakeholders. We have mapped out TSTC research evidence in Tanzania to inform on disease burden and potential for integrated control measures.

**Methodology/Principal findings:**

A scoping review of all TSTC studies undertaken in Tanzania and published up to December 2018 was conducted. The articles were searched from PUBMED, AJOL, Google Scholar and Google in general. Fifty-one (51) articles met the inclusion criteria and were reviewed. Prevalence of taeniosis of 2.3% - 5.2% was estimated based on copro-antigen ELISA while human cysticercosis of >16% was estimated based on serum antigen ELISA (Ag-ELISA) or IgG Western Blot. Neurocysticercosis (NCC) contributed significantly to epilepsy in adults. Farm prevalence of porcine cysticercosis were 6.0% - 17.4% (lingual examination) and 1.5% - 33.3% (Ag-ELISA). Slaughter-slab prevalence were 0% - 18.2% (routine meat inspection). Lacking latrines, watering pigs with river or pond water, and feeding pigs with potato peels were associated with porcine cysticercosis prevalence. Washing hands by dipping method increased the risk of human cysticercosis. In 2012, the number of DALYs/1000 person-years for NCC-associated epilepsy was 0.7 (95% UI, 0.2–1.6), around 5 million USD (95% UI, 797,535–16,933,477) were spent due to NCC-associated epilepsy and nearly 3 million USD (95% UI, 1,095,960–5,366,038) were potentially lost due to porcine cysticercosis. Three rounds of annual treatment of school-age children with praziquantel significantly reduced prevalence of taeniosis and porcine cysticercosis. Health education was efficacious in improving knowledge and attitudes favourable for control of TSTC while a single dose of oxfendazole 30 mg/kg body weight was efficacious in eliminating *T*. *solium* cysticerci from pig musculature.

**Conclusions/Significance:**

The observed high burden of TSTC and the significant contribution of NCC to epilepsy in Tanzania warrant urgent interventions. Evaluation of best control options should make use of disease transmission dynamics models such as cystiSim, taking into account findings from the field based intervention studies. In addition, locally adapted management guidelines for people suffering from NCC are urgently needed.

## Introduction

*Taenia solium* is a zoonotic tapeworm causing taeniosis in human (intestinal dwelling of an adult parasite) and cysticercosis in pig and human (tissue invasion with the larval form of the parasite). The parasite causes dual impact [1] because of infections in both hosts, posing considerable financial losses, mortalities especially in people with neurocysticercosis, morbidities and associated stigma, constituting disease burden in the affected communities [2]. *T*. *solium* taeniosis/cysticercosis (TSTC) has a worldwide distribution, and is endemic in many developing countries of Latin America, Asia, and Africa. Nevertheless, the increased globalisation enables TSCT to cross borders, calling for joint efforts in the control and ultimately eradication of the parasite.

In 2010, the World Health Organization (WHO) added TSTC to the list of Neglected Tropical Diseases (NTDs) requiring attention towards research, control and ultimately elimination [3]. In 2012, *T*. *solium* ranked first on the global scale of important food-borne parasites in terms of its impacts on public health and trade [4]. In 2013, the World Health Assembly passed the WHA66.12 resolution on NTDs, which promotes implementation of preventive and control strategies for taeniosis and cysticercosis in order to prevent epilepsy and other neurological and psychiatric disorders [5, 6]. Member countries were urged to ensure continued country ownership of programmes for NTD prevention, control and elimination, and to further strengthen the disease surveillance systems especially on NTDs targeted for eradication. The World Organization for Animal Health (OIE) published a cysticercosis code in 2015 to guide international trade on pigs and pig products from countries endemic for *T*. *solium* [7]. In Tanzania, TSTC has been identified as one of the country's important health research priorities, having been added to the list of the country's health research priorities for 2013–2018 and 2015–2020 [8, 9]. It is urged that research should focus on establishing the magnitude and trends of TSTC and device mechanisms for control [9].

Despite TSTC having been put high on the global agenda of neglected tropical diseases (NTDs), which over the last years has received a lot of attention [3–9], there has been no control programme in place in most endemic countries, Tanzania inclusive. This could be attributed to lack of awareness by many stakeholders of the burden and impact of the diseases. This information is essential in guiding TSTC policies, practices and research agendas as well as encouraging cross-sectoral collaboration in endemic countries. National elimination of the parasite is the foundation for global eradication. This will require substantial country-level information be provided to all key stakeholders [10], which is currently lacking in most TSTC endemic countries. In order to generate policy and/or practice changes, policy makers need a comprehensive overview of the situation at hand. This has not been established for TSTC in Tanzania and it cannot be provided by individual studies. The overall objective of the current study was to provide a research evidence base to guide country-level policies, practices and research agendas for control of TSTC in Tanzania. Specific objectives of the study were to provide information on the (1) magnitude, pattern and risk factors for TSTC in Tanzania, (2) public health and economic impacts of TSTC, (3) efficacy of tools for control of TSTC tested in Tanzania, (4) effectiveness of TSTC intervention strategies trialed in Tanzania, and (5) identify co-morbidities of TSTC infections in the country.

## Methods

### Inclusion criteria for studies

#### Types of participants

This review included all studies on TSTC in humans and pigs, including reviews.

#### Concept

The review included any study conducted to measure any aspect of TSTC.

#### Context/Types of studies

This review was intended to map the TSTC situation in Tanzania up to 31st of December 2018. Thus, only studies undertaken in Tanzania up to end of 2018 and addressing *T*. *solium* were included regardless of the setting (field or others) or study design. Both quantitative and qualitative studies were included. Studies that utilised biological samples from Tanzania for diagnostic test evaluation or other scientific purposes undertaken by researchers abroad were excluded from the review.

### Searching strategy

**Fig 1:**
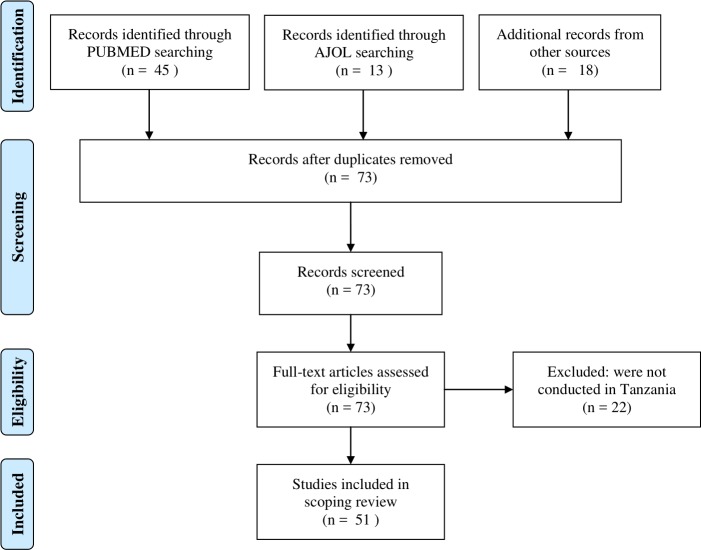
PRISMA diagram for a scoping review of *Taenia solium* taeniosis/cysticercosis research evidence in Tanzania, 1995–2018.

The literature searches were performed on February 7 and 8, 2019 and followed the standard three-steps as described in the Joanna Briggs Institute (JBI) guidelines [11] and an additional fourth step. In the first step, a decision was made on which databases to be included. Based on initial searches, PUBMED was found to be the most common database that captured most of the articles in this research topic. In addition, we included the African Journal Online (AJOL) to capture possible additional publications published in African journals. The second step was searching the two databases, performed by HAN. A full description of the PUBMED search is presented in the supporting information "S2 Table". The final search keywords were (((((("Taenia solium"[Mesh]) OR Pork tapeworm) OR Pork tapeworms) OR Tapeworm, Pork) OR Tapeworms, Pork)) AND (((("Tanzania"[Mesh]) OR United Republic of Tanzania) OR Zanzibar) OR Tanganyika) Filters: Publication date to 2018/12/31. Two entry terms ("Taenia soliums and soliums, Taenia) were removed from the search as PUBMED did not find "soliums" in its database. Similar keywords were used to search the AJOL database. Independently, HAN and EMM read through all the PUBMED and AJOL retrieved articles, one-by-one and selected those that, reported studies on TSCT conducted in Tanzania. In the third step, HAN searched all additional relevant articles cited in the list of references of each of the initially selected articles. These were also scanned through for additional references in their list of references. The fourth step involved an additional author-specific search in which persons known to have been involved in TSTC research in Tanzania were searched using Google Scholar and Google in general, which were also able to capture potential grey literature. The selection of articles for inclusion from the any search list was done consistently by HAN and EMM based on the agreed inclusion and exclusion criteria. The Preferred Reporting Items for Systematic Reviews and Meta-Analyses (PRISMA) flow diagram for this study is presented in Fig 1, while the Preferred Reporting Items for Systematic reviews and Meta-Analyses extension for Scoping Reviews (PRISMA-ScR) checklist is presented in S1 Checklist. As per PRISMA-ScR item 8 requirement, we have presented the full electronic search strategy for PubMed database and outlined the strategies used in searching other sources used (S2 Table).

### Extracting and charting the results

Data extraction and charting was performed as described elsewhere [11]. Data extraction and charting in this review included some coding and analysis of basic information to help in quantifying required information. For example, determining the number of studies that have investigated a particular aspect of TSTC in the country. Basic information extracted from each article is summarized in S1 Table. This include the study site, author, publication year, study aims, study design, study population and sample size, outcome measure, and key findings. In addition, the analytical data extraction and charting enabled further mapping of TSTC disease prevalence, transmission risk factors, societal costs, co-morbidities, efficacy of disease intervention tools, and effectiveness of control strategies tested in the country. The literature review was performed in ATLAS.ti 8 by reviewer HAN and manually by EMM, and any disparity was sorted out in a scheduled meeting between the two reviewers. The charting results obtained by these reviewers were verified and agreed by all other authors who independently submitted their feedback to HAN.

## Results

### General results

**Fig 2:**
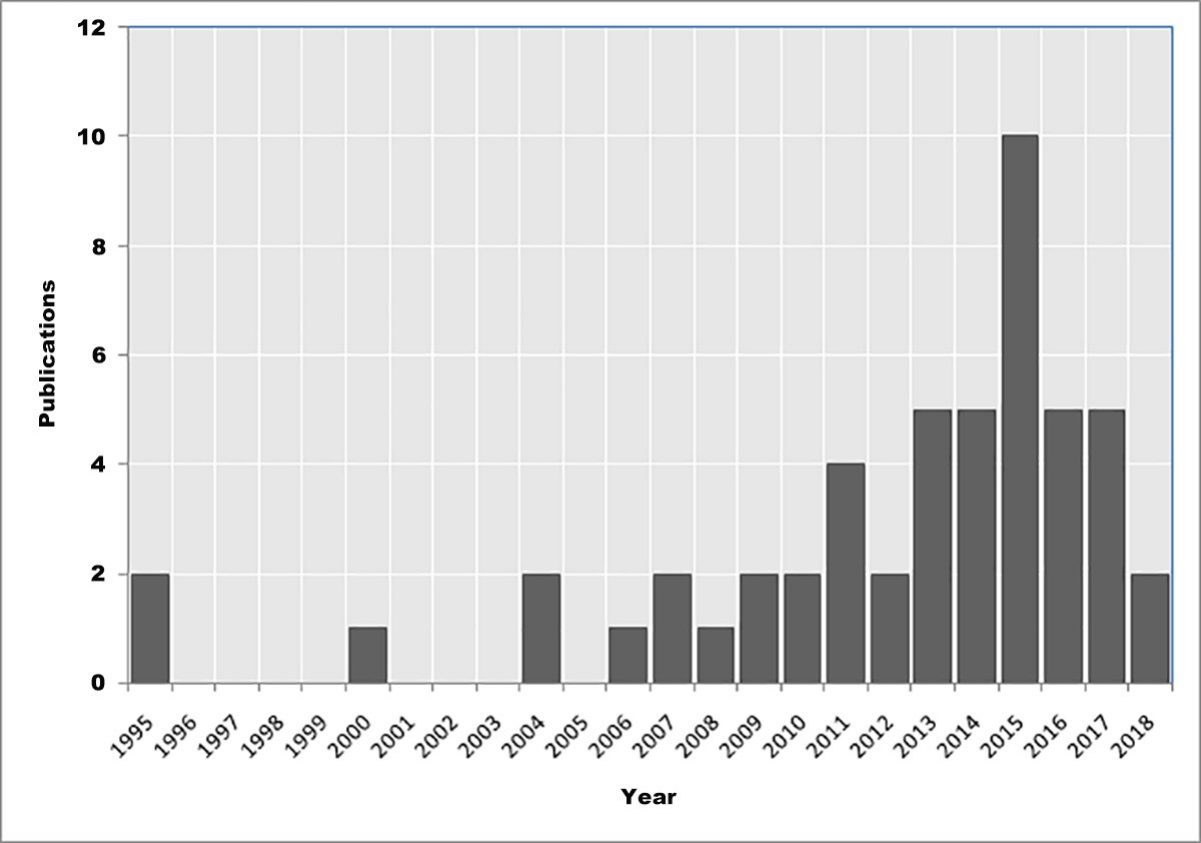
Number of *Taenia solium* publications per year in Tanzania, 1995–2018.

**Fig 3:**
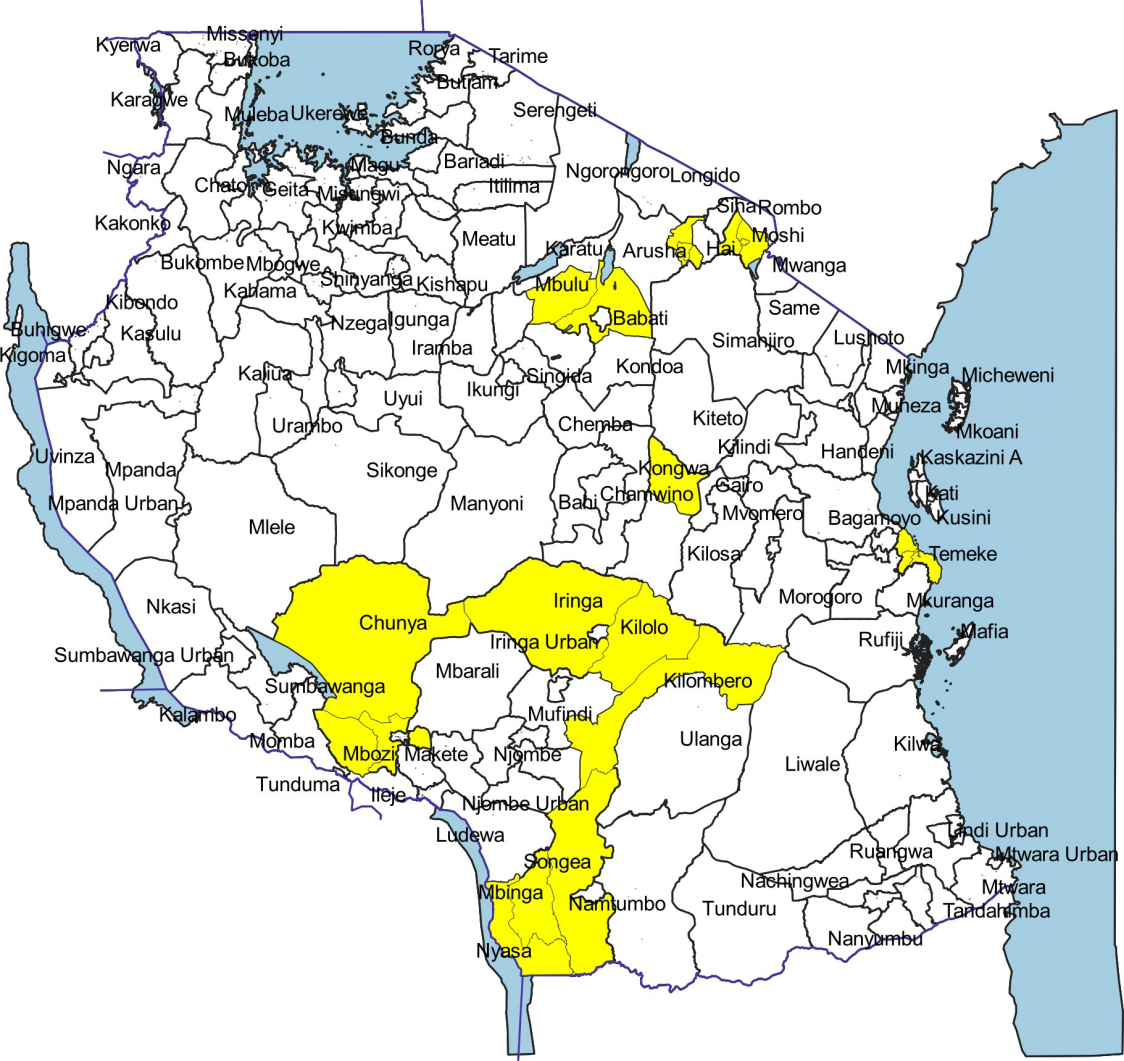
Locations (yellow) of previous studies for *Taenia solium* taeniosis/cysticercosis in Tanzania. 1995–2018.

**Fig 4:**
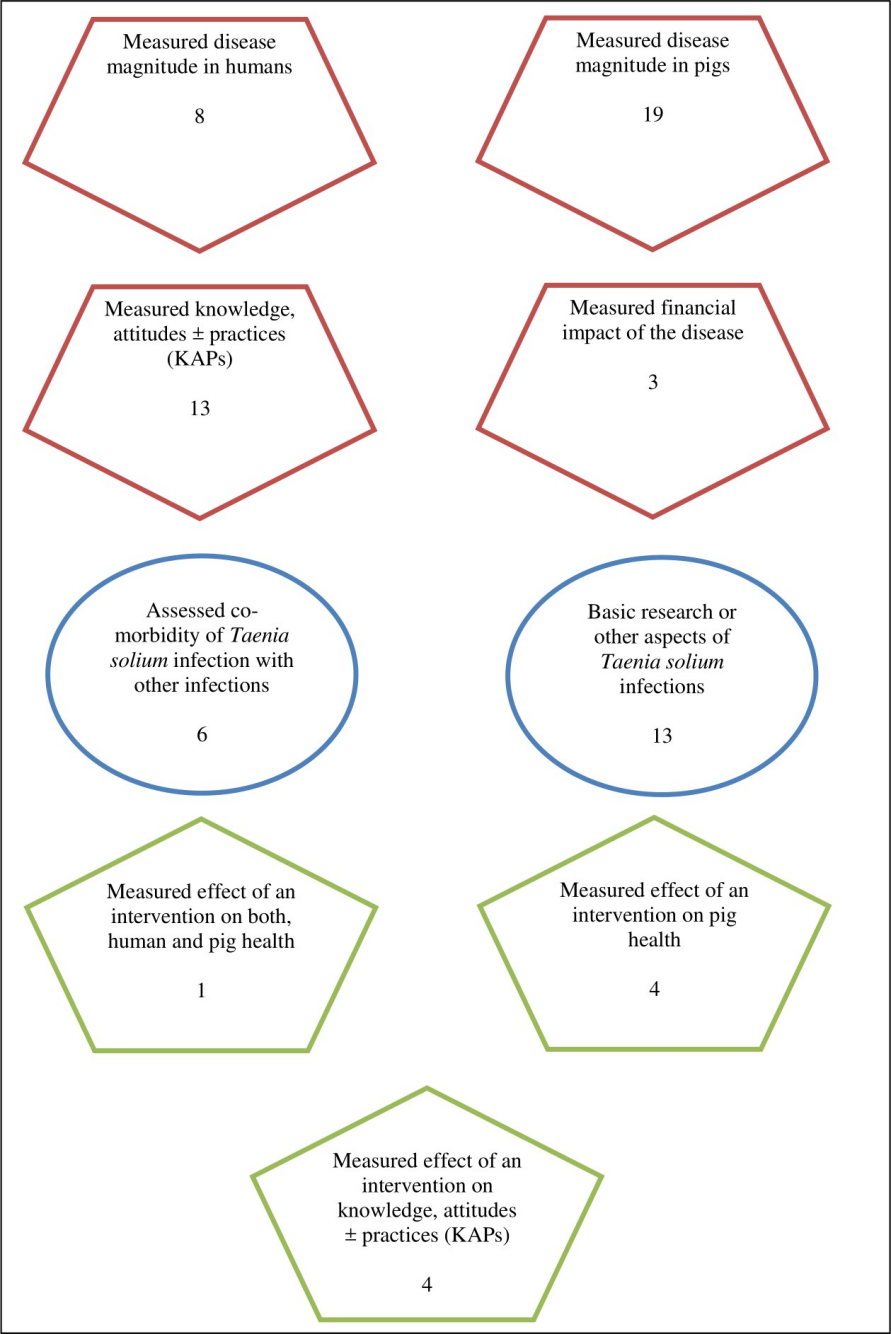
Number of previous *Taenia solium* taeniosis/cysticercosis studies by outcome measure in Tanzania, 1995–2018.

A total of 76 scientific articles were retrieved, of which 51 met the inclusion criteria for review. The reviewed articles were published from 1995 to December 2018 [12–62] (S1 Table). Of these, 47 were full-length journal papers [12, 14–27, 29–45, 47–60, 62], three were short communications [13, 28, 61], and one was a dissertation book [46]. There were more publications from 2007 on with peak publication in 2015 compared to previous years (Fig 2). Study sites for original investigations are mapped in Fig 3, showing clustering of studies in some parts of the country especially the southern and northern highlands. Fig 4 presents the number of studies by various aspects of TSTC investigated. The studies assessed disease burden for humans and pigs separately and only one study had measured effects of an intervention on human and pig health simultaneously [23].

### Prevalence, distribution and temporal pattern of *Taenia solium* infections

#### Human taeniosis and cysticercosis

Previous studies adopted various study designs, diagnostic tests, and target groups to estimate the morbidity of TSTC in humans. This scoping review presents the study results *in situ* without an attempt to compare levels of infections between studies, sites, or periods. Table 1 presents the prevalence of TSTC in humans based on studies conducted in general populations of the study districts. These studies have estimated prevalence of taeniosis ranging from 0.4% to 5.2% based on Kato-Katz or copro-antigen enzyme-linked immunosorbent assay (CoAg-ELISA), respectively [14, 16]. Cysticercosis of approximately 16–17% based on Ag-ELISA or IgG western blot methods were estimated [14, 27] (Table 1). Table 2 presents prevalence of NCC in people with and without epilepsy, and the association between epilepsy and NCC [14, 19, 25, 43, 54]. All but one study [43] examined adult populations (especially those > 15 years old) and found statistically significant association between epilepsy and NCC.

**Table 1:** Prevalence of human *Taenia solium* taeniosis/cysticercosis in the general populations in Tanzania from 1995–2018.

Reference	Study area	Study population and sample size	Diagnostic test	Taeniosis (%)	Cysticercosis (%)
[14]	Mbozi district	830 people 15–60 years old	CoAg-ELISA	5.2	
			Ag-ELISA		16.7
			Ab-ELISA		45.3
			Microscopy	1.1	
[16]	Kongwa district	1057 people	Kato-Katz	0.4	
[27]	Mbulu district	544 people	IgG western blot		16.3
[60]	Mbozi & Mbeya Rural districts	561 Adults >15 years old	CoAg-ELISA	4.1	
		951 Children < 16 years old	CoAg-ELISA	2.3	

**Table 2:** Prevalence of neurocysticercosis (NCC) in people with epilepsy (PWE) and without epilepsy (PWOE), Tanzania, 1995–2018.

Reference	Study area	Study population and sample size	Diagnostic test	Definitive NCC (%)	Association with epilepsy
[14]	Mbozi district	28 Ag-ELISA+ PWE 27 Ag-ELISA+ PWOE	CT scan	100 7.4	P < 0.0000
[19]	Kilombero district	300 PWE 300 PWOE	Western blot using cysticercosis antigen (rT24H) and taeniosis antigen (rES33)		Significant association in adults OR 7.03 (95% CI: 2.06, 24.00); P = 0.002
[25]	Mbulu district	212 PWE 198 PWOE	CT scan + Antibody detection	3.3	P < 0.0001
[43]	Kilombero district	278 PWE 345 PWOE	Western blot using rT24H and rES33 antigens		No association
[54]	Hai district	218 PWE 178 PWOE	Western blot using rT24H and rES33 antigens	1.1	Association with Adult PWE : P = 0.04

#### Porcine cysticercosis

As with human studies, porcine studies adopted various study designs, diagnostic tests and target groups to estimate the prevalence or incidence of porcine cysticercosis. Table 3 presents prevalence of porcine cysticercosis reported in Tanzania [12, 13, 15, 17, 18, 20, 21, 24, 26, 32, 46, 53, 56–58, 62]. Pig-farm based studies have reported prevalence ranging from 6.0% - 17.4% based on the lingual examination method [12, 15, 21, 24, 32, 53] and 1.5% - 33.3% based on Ag-ELISA [12, 13, 15, 26, 46, 56, 57] (Table 3). One incidence rate study in northern Tanzania estimated incidence rate of 25 per 100 pig-years based on lingual examination and 69 per 100 pig-years based on Ag-ELISA in sentinel pigs [18]. On the other hand, slaughter-slab studies have reported prevalence of porcine cysticercosis in slaughter pigs ranging from 0–18.2% based on routine meat inspection [12, 17, 20, 26, 58, 62] (Table 3). A longitudinal study composed of three cross-sectional surveys in Mbeya Region revealed temporal fluctuation of porcine cysticercosis seroprevalence [57].

**Table 3:** Prevalence of porcine cysticercosis on pig farms and at slaughter slabs in Tanzania, 1995–2018.

Reference	Study area	Sample size	Prevalence on pig farms (%)		Prevalence at slaughter slabs (%)
			Lingual exam (%)	Ag-ELISA (%)	Meat inspection (%)
[12]	Nyasa district	698, 22, 330 for tongue, meat inspection and Ag-ELISA, respectively	6.3	33.3	18.2
[13]	Mbozi and Mbeya Rural districts	482		11.5	
[15]	Mbozi district	300	11.7	32	
	Mbeya Rural district	300	6.0	30.7	
[17]	Dar es Salaam city	731			5.9
[18]	Mbulu district	Pig-months of follow up in the control group was 690 and 594 by lingual and Ag-ELISA, respectively	25/ 100 pig-years Incidence rate	69/100 pig-years Incidence rate	
[20]	Mbulu, Arusha and Moshi towns	70			0
[21]	Mbulu	770	17.4		
[24]	Chunya district	722	7.6		
	Iringa Rural	808	8.4		
	Mbinga district	302	16.9		
[26]	Babati district	442 live pigs and 1039 pig carcasses	13.0	25.0	8.2
[32]	Kongwa district	309	14.9		
[46]	Morogoro district	260		1.5	
[53]	Iringa Rural district	308	7.5		
[56]	Mbozi and Mbeya Rural districts	142		26.0	
[57]	Mbozi district	822, 812, 998 baseline, 6 and 14 months reassessment		15.0, 24.0, 20.0, respectively	
[58]	Mbulu, Arusha and Moshi towns	83			13.3
[62]	Arusha, Dar es Salaam and Mbeya cities				1.74, 6.3, 0.27, respectively

### Risk factors for *Taenia solium* infections

Risk factors that have been found to significantly be associated with high prevalence of porcine cysticercosis are free range husbandry systems [12, 15, 26, 32], lacking latrine in the household [12, 21], sourcing water for drinking from rivers or ponds [15], and feeding pigs on potato peels [39]. Only one study had assessed risk factors for human infection. The study found that hand washing by dipping (instead of running water) was significantly associated with Ag-ELISA seropositivity in humans [14].

### Societal impacts of *Taenia solium* infections

Only three studies had attempted to estimate economic implications of *T*. *solium* infections. One of the studies analysed the financial benefit to smallholder pig farmers in Mbulu district, northern Tanzania, of attending a health education training to control porcine cysticercosis. The study found that over a 5-year period, a health education intervention had significant financial benefit to smallholder pig farmers [net present value: US $3507 (95% CI: 3421 to 3591); internal rate of return: 370%] [30]. Another study estimated pig farmers' perceived financial loss due to porcine cysticercosis and human epilepsy in Iringa Rural district [31]. The authors estimated an annual monetary loss due to porcine cysticercosis of USD 144,449 and an annual monetary burden due to epilepsy management in hospitals and/or by traditional healers of USD 78,592. Subsequently, a comprehensive systematic review of the available literature was carried out to estimate societal cost of *T*. *solium* cysticercosis in Tanzania [34]. The study found that for the year 2012 the number of DALYs per thousand person-years for NCC-associated epilepsy was 0.7 (95% UI, 0.2–1.6). Around 5 million USD (95% UI, 797,535–16,933,477) were spent due to NCC-associated epilepsy and nearly 3 million USD (95% UI, 1,095, 960–5,366,038) were potentially lost due to porcine cysticercosis [34].

### Co-morbidity of *Taenia solium* infections with other health problems

Seven studies assessed co-morbidity of *T*. *solium* infection with other infections in Tanzania [14, 25, 32, 37, 40, 43, 54] (Table 4). Three of four studies that assessed potential contribution of NCC to epilepsy in humans reported that NCC was a significant a epilepsy associated with epilepsy in adult people [14, 25, 54]. Additional aspects of TSTC co-morbidity with other infections are presented in Table 4. However, these involved single studies, which makes it impossible to draw firm conclusions regarding the reported associations. For example, one study in pigs found co-morbidity of porcine cysticercosis, trichuriosis and strongyle worms [32].

**Table 4:** Co-morbidity of *Taenia solium* infections with other infections in Tanzania, 1995–2018.

Reference	Study population	*Taenia solium* infection assessed	Co-morbidity with	Key findings
[25]	212 PWE and 198 PWOE at hospital	Human neurocysticercosis based on serology and CT scanning	Epilepsy	NCC lesions were significantly more frequent in people with epilepsy compared to controls (p < 0.0001).
[14]	Ag-ELISA PWE and Ag-ELISA PWOE from community	Human neurocysticercosis based on serology (Ag-ELISA) and CT scanning	Epilepsy	All of the 28 Ag-ELISA positive people with a history of epileptic seizures were CT-scan positive for NCC while only two of the 27 Ag-ELISA positive people without epilepsy were CT-scan positive for NCC.
[43]	278 PWE and 345 age-matched PWOE from community	Human taeniosis/cysticercosis antibody seroconversion	Epilepsy	The prevalence of *T*. *solium* antibodies was low (2.8% of cases and 2.2% of controls) and was not associated with active convulsive epilepsy.
[54]	Adult PWE and PWOE from community	Neurocysticercosis	Epilepsy	Six of 218 PWE had antibodies to *T*. *solium* compared to none of 174 controls (P = 0.0137). Lesions compatible with NCC were seen in eight of 200 CT scans (4.0%; 95% CI 1.3–6.7)
[37]	170 HIV+ and 170 HIV- controls humans	Taeniosis, Cysticercosis, and Neurocysticercosis	HIV/AIDS	No significant differences between HIV+ and HIV–individuals regarding the sero-prevalence of taeniosis antibodies, cysticercosis antibodies/antigens or CT scan NCC lesions.
[32]	Rural pigs	Porcine cysticercosis lingual cysts	GIT helminths	None of 36 pigs infected with cysticercosis had ascariosis, one had trichuriosis and seven had strongyle worm infections. Geographically, there was inverse occurrence between porcine cysticercosis and GIT helminth infections, with porcine cysticercosis preferring villages practising free-range while GIT helminths prefer areas practising pig confinement.
[40]	Slaughter pigs	Porcine cysticercosis at meat inspection	*Taenia hydatigena* cysts	Co-infections were not observed during this study. Co-endemicity was found in which 16 pigs were infected with *T*. *hydatigena* while two were infected with *T*. *solium*.

### Efficacy of *Taenia solium* control tools in Tanzania

Four of the published studies assessed efficacy of an intervention tool for control of TSTC infection in humans and/or pigs [28, 29, 45, 55] (Table 5). A health education intervention study based on educating pig farmers significantly improved their knowledge and attitude favourable to the control of TSTC [28]. Similar results were obtained from a school-based cluster-randomised trial [55]. In addition, an electronic educational tool, The Vicious Worm, was found to be efficacious in improving knowledge of veterinary and health professionals regarding TSTC [45]. One porcine cysticercosis treatment trial proved the efficacy of a single dose of oxfendazole 30 mg/kg body weight in clearing *T*. *solium* cysticerci in the pig musculature but not those located in the brain [29].

**Table 5:** Efficacy of *Taenia solium* control tools previously evaluated in Tanzania, 1995–2018.

Reference	Study area	Intervention	Target for intervention	Population evaluated	Key findings
[29]	Sokoine University of Agriculture	Randomised parallel groups: GP1—Subcutaneous injection of ivermectin at 0.3 mg/kg body weight GP2—Oral administration of oxfendazole at 30 mg/kg body weight GP3—Monitoring alone	Pigs	Pigs	Ivermectin had no effect on *T*. *solium* cyst viability. Oxfendazole had significant effect on cyst viability (p < 0.001) in all muscle tissues except brain. Both drugs significantly reduced faecal egg count of roundworms (p < 0.001). Ivermectin was 100% effective in control of mange caused by *Sarcoptes scabiei*.
[28]	Iringa Rural and Chunya districts	A health education package consisting of (1) training of trainers (livestock extension agents, (2) an address to pig farmers by a trainer, (3) a video show, and (4) distribution of a leaflet and a comic booklet to each participant.	Pig farmers	Pig farmers	Health education intervention significantly improved knowledge and attitudes towards *Taenia solium* control (P < 0.001)
[55]	Mbulu district	A health education package consisting of (1) training of trainers (school teachers), (2) an address by the trainer to children, (3) a video show and (4) distribution of one leaflet to each participant.	School children (primary and secondary schools)	School children	The overall score (percentage of correct answers) improved by about 10% in all schools after 6 months. Monitoring alone was associated with improvement in scores by about 6%. The intervention was linked to improvements in the attitude of condemning infected meat. The intervention reduced the attitude of contacting a veterinarian if a pig was found to be infected with cysticercosis.
[45]	Mbeya town	The Vicious Worm, an electronic educational tool for TSCT	Medical and Veterinary professionals	Medical and Veterinary professionals	Knowledge was significantly improved both immediately after (p = 0.001) and two weeks after (p<0.001) the intervention.

### Effectiveness of *Taenia solium* control strategies in Tanzania

Three studies assessed TSTC preventive effectiveness under field conditions [18, 22, 23] (Table 6). A village-level randomised health education intervention trial in 42 villages in northern Tanzania found out that the health education reduced the incidence rate of porcine cysticercosis by approximately 43% [18]. However, the education did not improve pig confinement or use of latrines. Another cluster-randomised trial assessing effectiveness of integrated pig management intervention programme (improved housing + improved feeding + oxfendazole treatment) found no significant effect of the intervention on porcine cysticercosis, though it significantly prevented ectoparasites and some gastrointestinal helminths of the pigs [22]. The only study that assessed the effect of a TSTC intervention on infections in humans and pigs simultaneously revealed that three-rounds of annual mass drug administration of praziquantel to school-age children (primarily targeted for schistosomiasis control) combined with ‘track-and-treatment’ of cases, significantly reduced the prevalence of taeniosis in children and adult populations as well as porcine cysticercosis [23]. However, two rounds of intervention were ineffective in producing a significant drop in porcine cysticercosis. Overall, there is limited information from previous studies regarding cost-effectiveness of TSCT control options.

**Table 6:** Effectiveness of *Taenia solium* control strategies evaluated in Tanzania, 1995–2018.

Reference	Study area	Intervention	Target for intervention	Population evaluated	Key findings
[23]	Mbozi district	Three rounds of annual mass drug administration of praziquantel, targeting control of schistosomiasis combined with ‘track-and-treat’	School-age children	General population Pigs	Significantly fewer children were infected throughout the study based on copro-Ag-ELISA. During the final survey, prevalence of taeniosis in adults (1.8%) was significantly lower (p = 0.031, OR 0.40, CI: 0.17–0.89), compared to baseline (4.1%). The prevalence of porcine cysticercosis (8%) had also dropped significantly (p = 0.002, OR 0.49, CI: 0.32–0.76) compared to baseline (13%),
	Mbeya Rural district	Two rounds of annual mass drug administration of praziquantel, targeting control of schistosomiasis combined with ‘track-and-treat’	School-age children	General population Pigs	Significantly fewer children were found infected after the first treatment only. No significant drop in porcine cysticercosis.
[18]	Mbulu district	A health education package consisting of (1) training of trainers (livestock extension agents, (2) an address to pig farmers by a trainer, (3) a video show, and (4) distribution of a leaflet and a comic booklet to each participant.	Smallholder pig farmers, livestock and health extension agents	Smallholder pig farmers	A reduction in the incidence rate of porcine cysticercosis of approximately 43% by the intervention. There was no significant effect of the intervention on knowledge as both groups improved significantly after intervention. The intervention did not bring about any significant improvement in pig confinement or use of latrine.
[22]	Mbozi and Mbeya Rural districts	The following combination: a) specific training and technology transfer of improved pig pens,(with demo pens) b) improved pig feeds and feeding practices, and c) treatment with oxfendazole for cysticercosis	Pig farmers. Pigs	Pigs	The intervention did not have any significant effect on the prevalence of porcine cysticercosis. The intervention reduced the prevalence and burden of roundworms and ectoparasites (P < 0.05)

## Discussion and conclusions

### Discussion

This review has revealed increasing research in TSTC in Tanzania over time, though clustered in some parts of the country, particularly in the southern and northern highland regions. While several studies have estimated disease morbidity, a negligible number have estimated economic impact of the parasite. The only reliable study in this aspect is one systematic review conducted in the country [34]. Similarly, very few studies have measured the effect of TSTC interventions on disease morbidity, with only one measuring intervention effect on human and pig disease simultaneously within the same area [23]. In addition, there is limited information to inform on spatial pattern of infections countrywide due to the clustered nature of the studies, covering only some few parts of the country. The observed higher prevalence of porcine cysticercosis during dry season [57] need further investigation, though it is customary for pigs to be let free during dry season to a larger degree than during the wet season, which could partly account for the observed differences. The fact that only one wet season as opposed to two dry seasons was assessed, no firm conclusion can be drawn from these findings in relation to seasonal pattern of TSTC infections.

Previous studies in Tanzania have detected taeniosis in the general population based on both antigen detection and microscopy of human faecal material. As both of these methods cannot identify *Taenia* eggs to species level, the possibility exist that some of the reported taeniosis cases are due to *T*. *saginata*, the beef tapeworm. One molecular study confirmed that one of four *Taenia* egg positive cases was due to *T*. *solium* [16]. The reported prevalence of human cysticercosis of more than 16% based on antigen detection in the general population is alarming. Furthermore, the significant association between epilepsy and NCC in adults found by most studies is consistent with findings from studies elsewhere [63]. Washing hands by dipping instead of using running water puts the person at a significant risk of contracting cysticercosis [14]. The prevalence of porcine cysticercosis estimated by previous studies by lingual examination and Ag-ELISA both show that porcine cysticercosis is endemic in Tanzania. Free range pig farming [12, 15, 26, 32], lack of latrines in the household [12, 21], providing pigs water from rivers or ponds [15], and feeding pigs potato peels [39] have been associated with high prevalence of porcine cysticercosis. Health education could help in improving practices to the control of TSTC transmission.

One intervention study has revealed that the annual schistosomiasis control programme involving treatment of school-age children using praziquantel significantly reduced prevalence of taeniosis in children and adult populations as well as porcine cysticercosis if at least three rounds of annual mass deworming are conducted [23], suggesting the potential for integrated control of these NTDs. One main drawback of this pre-post intervention evaluation study is the lack of a control group for ethical reasons. Thus the study is unable to link the observed changes in the disease frequency with the interventions. In addition, a note of caution with regards to potential neurological side effects has to be added as in those areas that are co-endemic with schistosomiasis and TSCT latent NCC may be exacerbated by treatment of schistosomiasis with praziquantel (unpublished data, AS Winkler). A village-level randomised health education intervention study found a reduction in the incidence rate of porcine cysticercosis of approximately 43% attributable to the intervention [18]. Further analysis proved financial efficiency of the health education intervention to the smallholder farmer receiving it [30]. Nevertheless, the health education could not improve pig confinement or use of latrines [18]. It is speculated that, farmers might have changed some other important but unobservable practices, which could partly explain the observed reduction in the incidence rate of porcine cysticercosis. One important limitation of this study is lack of evaluation of the effect of the intervention on human prevalence of taeniosis. Finally, one porcine cysticercosis treatment trial proved the efficacy of oxfendazole 30 mg/kg body weight in clearing *T*. *solium* cysticerci in the pig musculature but not those in the brain, which is consistent with findings from other studies elsewhere [29]. In summary, each of the previous TSTC intervention studies conducted in Tanzania had one or more study-design related limitation(s), including lack of randomisation, absence of a control group or evaluation of intervention effect to only one of the two hosts (pig or human). These findings are consistent with a worldwide literature review of TSTC intervention studies [64]. Thus, at present, although different tools have proven efficacious in reducing prevalence in either pigs or humans, community based One Health cost-effectiveness studies are now highly warranted to determine the best control options. To assist in developing effective control/elimination programmes in Tanzania and elsewhere, disease transmission dynamics models (e.g. cystiSim) [36] could be used. Several TSTC transmission dynamics models have been developed [36, 65, 66].

The only limitation of this review is the possibility of having missed unpublished information regarding TSCT in Tanzania because of the study design. However, the possibility is considered minimal due to the fact that research on TSCT in the country to date has been mostly undertaken by few institutions whose researchers have been active publishers of their research works. This limitation is further narrowed down by our inclusion of the fourth step search that targeted specific TSTC researchers who have been commonly involved in TSTC research in Tanzania. Nevertheless, we still admit that there could be some chance of a few TSTC research in Tanzania remaining in offline repositories (e.g. some institutional libraries) as hard-copy publications, which would not be captured by our online search strategy.

### Conclusions

This review has gathered research evidence that confirms TSTC is a serious problem of public health and economic importance in Tanzania, and calls for urgent control measures to be implemented. The use of disease transmission dynamics models could help in suggesting best control strategies to be evaluated in Tanzania, taking into considerations findings from the field based intervention studies. In addition, suitable guidelines for managing people suffering from NCC are urgently needed.

### Implications for research and practice

The observed considerable societal burden of TSTC and significant contribution of NCC to epilepsy in Tanzania warrants an urgent intervention to safeguard public health and improve livelihoods. More studies are needed to better estimate cost-effectiveness of TSTC control options in order to implement cost-beneficial TSTC control measures in Tanzania.

## Supporting information

S1 Checklist PRISMA-ScR items for *Taenia solium* evidence in Tanzania, 1995–2018. (DOC)

S1 Table Characteristics of studies reviewed for *Taenia solium* in Tanzania,1995–2018. (DOC)

S2 Table Fill list of terms searched for *Taenia solium* evidence in Tanzania,1995–2018. (DOC)
