# Supplementary material for: Taenia solium taeniosis and cysticercosis literature in Tanzania provides research evidence justification for control: A systematic scoping review
Source: PLoS One. 2019 Jun 5;14(6):e0217420. doi: 10.1371/journal.pone.0217420 (PMC6550401; doi:10.1371/journal.pone.0217420)
Supplement: S1 Table — (DOC) [file pone.0217420.s002.doc]

| **Reference** | **Publication year** | **Study sites (districts. Otherwise stated)** | **Aims of study with respect to *Taenia solium*** | **Study design (field, otherwise stated)** | **Study population and sample size** | **Outcome measure(s)** | **Key findings** |
| --- | --- | --- | --- | --- | --- | --- | --- |
| [12] | 2017 | Nyasa | Estimation of prevalence of porcine cysticercosis and transmission risk factors | Cross-sectional | 698 pigs tongue exam; 330 pigs Ag-ELISA; 22 pig-meat inspection; Questionnaire respondents ?n | Prevalence | Porcine cysticercosis prevalence by tongue examination 6.3% (95% C.I. 4.5–8.1%), by Ag-ELISA 33.3%, (95%C.I. 28.22–38.38%) and meat inspection 18.2% (95% C.I. 2.08–34.32%). Risk factors: free ranging of pigs (p = 0001); outdoor defecation (0.0001). |
| [13] | 2015 | Mbozi; Mbeya Rural | Estimation of prevalence of porcine porcine cysticercosis and transmission risk factors | Cross-sectional | 482 pigs; 220 respondents | Prevalence | Porcine cysticercosis prevalence 11.5% (95% CI: [8.8–14.7]) |
| [14] | 2013 | Mbozi | Estiimation of prevalence of *T.solium* infections in humans and transmission risk factors | Cross-sectional | 830 humans tested | Prevalence | Human Ag-ELISA seroprevalence 16.7%; Ab-ELISA seroprevalence 45.3%; 54.6% of Ag-ELISA positive individuals had structures in the brain suggestive of NCC by CT scan; Faecal CoAg-ELISA seroprevalence was 5.2% while microscopy was 1;1%; Hand washing by dipping in contrast to running water, increased Ag-ELISA seropositivity |
| [15] | 2013 | Mbozi; Mbeya Rural | Estimation of prevalence of porcine cysticercosis and transmission risk factors | Cross-sectional | 600 pigs; 300 respondents | Prevalence | Porcine cysticercosis prevalence in Mbozi district 11.7% (95% CI = 8.5–15.8%) and 32% (95% CI: 27–37.5%) based on lingual examination and Ag-ELISA, respectively; In Mbeya Rural district, 6% (95% CI: 3.8–9.3%) and 30.7% (95% CI:25.8–36.1%), respectively; Important risk factors: free roaming of pigs (OR = 2.1; 95% CI = 1.3–3.6; p = 0.006), in-house origin of the pig (OR = 1.6; 95% CI = 1.1–2.5) and sourcing of water from rivers(OR = 3.1; 95% CI = 1.6–6.3; p < 0.001) and ponds (OR = 5.0; 95% CI = 1.2–21.7; p = 0.031). |
| [16] | 2011 | Kongwa | Morphological and genetic identification of Taenia tapeworms | Cross-sectional (2 years) | 1057 humans samples | Prevalence | 4 taeniasis cases were found 1 of which was confirmed to be *T. solium* |
| [17] | 2010 | Kinondoni, Ilala & Temeke | Estimiiation of prevalence of porcine cysticercosis in slaughter pigs | Cross-sectional | 731 pigs | Prevalence | Porcine cysticercosis prevalence 5.9%. Almost all slaughter pigs in Dar es Salaam originated from other regions, endemic for porcine cysticercosis |
| [18] | 2008 | Mbulu | Assessment of effectiveness of health education intervention on incidence rate of porcine cysticercosis | Cluster-randomised controlled trial | Pig-months of follow up Ag-ELISA 651 & 594 in intervention and control groups, respectively. Lingual exam 722 & 690, respectively; 799 respondents | Incidence rate; Incidence rate ratio | incidence rate of porcine cysticercosis in the control group 10–12 months after the intervention was 69 (95% CI: 65, 72) per 100 pig-years and 25 (95% CI: 23, 28) per 100 pigyears, using Ag-ELISA and lingual examination, respectively. The incidence rate ratio comparing the intervention group to the control group and adjusting for the baseline prevalence of porcine cysticercosis were 0.57 (95% BCI: 0.3, 1.0), a reduction of approximately 43% by the intervention; There was no signiﬁcant effect of the intervention on knowledge as both groups mproved their knowledge signiﬁcantly. The intervention did not have any signiﬁcant improvement pig confinement or latrine use. |
| [19] | 2013 | Ifakara | Assessment of contribution of *T. solum* to epilepsy | Cross-sectinal & Case-Control | 104,889 humans | Odds ratio | In adults (aged ≥18 years),epilepsy was significantly associated with exposure to T solium (OR 7•03, 2•06–24•00; p=0•002) |
| [20] | 2004 | Mbulu; Arusha; Moshi | Estimiiation of prevalence of porcine cysticercosis in slaughter pigs | Cross-sectional | 70 pig carcasses | Prevalence | Porcine cysticercosis was not detected |
| [21] | 2004 | Mbulu | Estimation of prevalence of porcine cysticercosis and transmission risk factors | Cross-sectional | 770 pigs; 436 respondents | Prevalence | Porcine cysticercosis prevalence 17.4% based on lingual exam; Prevalence was considerably higher in pigs reared in households lacking latrines (OR = 2.04; 95% BCI = 1.25, 3.45). About 96% of the pigs were kept under free-range system or let to roam at some point of their life |
| [22] | 2018 | Mbozi; Mbeya Rural | Assessment of effectiveness of integrated pig management intervention programme in the control of pig parasites | Cluster-randomised controlled trial | 482, 460, 421 pigs at baseline,1st and 2nd follow-up, respectively | Change from baseline prevalence | No signiﬁcant effect of the intervention on porcine cysticercosis based on Ag-ELISA (p > 0.05). The intervention signiﬁcantly reduced prevalence of *Trichuris suis,* mean faecal egg counts of *Ascaris suum* and prevalence of ectoparasites (p<0.05) |
| [23] | 2016 | Mbozi; Mbeya Rural | Assessment of effect of national schistosomiasis (praziquantel treatment) progamme in the control of *T. solium* infections in humans and pigs | Repeated cross-sectional | 12,082 humans tested | Change from baseline prevalence | Signiﬁcantly fewer children from Mbozi (where 3 rounds of MDA were given) were infected throughout the study than children from Mbeya (where only 2 rounds were given) who showed a signiﬁcant decrease in copro-Ag prevalence after the ﬁrst treatment only. During the ﬁnal survey in Mbozi the prevalence of taeniosis in adults (1.8%) was signiﬁcantly lower (p = 0.031, OR 0.40, CI: 0.17–0.89), compared to baseline (4.1%). The prevalence of porcine cysticercosis (8%) had also dropped signiﬁ- cantly (p = 0.002, OR 0.49, CI: 0.32–0.76) compared to baseline (13%), whereas no signiﬁcant difference was seen in Mbeya. |
| [24] | 2006 | Chunya; Iringa Rural; Mbinga | Estimation of prevalence of porcine cysticercosis and transmission risk factors | Cross-sectional | 722, 808 and 302 pigs respectively | Prevalence | Prevalence of porcine cysticercosis were 7.6%, 8.4% and 16.9% in Chunya and Iringa Rural Districts, and Ruvuma Region, respectively. |
| [25] | 2009 | Mbulu | Assessment of contribution of *T. solum* to epilepsy | Case-Control (hospital based) | 212 humans with epilepsy; 198 without epilepsy | Odds ratio | NCC lesions were signiﬁcantly more frequent in people with epilepsy compared to controls (p < 0.0001). CT results, and serum and CSF analysis taken together, we diagnosed 22 (10.4%%) individuals with probable and 7 (3.3%%) with deﬁnitive NCC in our cohort of people with epilepsy. |
| [26] | 2017 | Babati | Estimation of prevalence of porcine cysticercosis and transmission risk factors | Cross-sectional | 442 pigs; 150 respondents | Prevalence | Prevalence of porcine cysticercosis 13%, by lingual exam and 25% by Ag-ELISA test. Abattoir survey found a prevalence of .8.2%. Free ranging of pigs was a significant risk factor (p = 0.0002) for porcine cysticercosis |
| [27] | 2012 | Mbulu | Estimation of *T. solium* antibody seroprevalence in the general population | Cross-sectional | 544 humans tested | Prevalence | Approximately, 16.3% of the community members tested had antibodies against *T. solium* cysticercosis |
| [28] | 2011 | Iringa Rural; Chunya | Assessment of effectiveness of health education intervention on *T. Solium* knowledge and attitudes | Quasi-Experiment | 750 & 700 respondents in Iringa rural & Chunya districts, respectively | Change from baseline prevalence | Health education intervention significantly improved the knowledge and attitudes towards *Taenia solium* control (P < 0.001) |
| [29] | 2013 | SUA experiment | Assessmet ofo efficacy of ivermect and oxfendazole administered parallel in pigs naturally infected with cysticercosis | Randomised controlled experiment (SUA) | 61 pigs | Difference between means | Ivermectinhad no signiﬁcant effect (p = 0.224) on *T. solium* cysts viability compared to the control group. Oxfendazole had significant effect on cyst viability (p < 0.001) compared to ivermectin and control groups in all muscle tissues. None of the drugs was effective in brain cysts. Both drugs signiﬁcantly reduced (p < 0.001) faecal egg count of *Ascaris suum*, strongyles and *Trichuris suis* two weeks after treatment. At slaughter, Oesophagostomum dentatum, Ascarops strongylina and Physocephalus sexalatus were recovered from pigs in the IVM treated and in the control groups. Ivermectin was 100% effective in control of Sarcoptes scabiei. |
| [30] | 2007 | Mbulu | Assessment of financial efficiency of health education intervention on porcine cysticercosis control | Investment appraisal | 36 key informants | Net present value; Internal rate of return | Over a period of 5 years, the health education intervention would have a significant financial benefit to the smallholder pig farmers in Mbulu District [NPV: US $3507 (95% CI: 3421 to  3591); IRR: 370%]. The intervention would remain financially efficient regardless of plausible changes in costs and benefits in the pig production, as well as plausible changes in the incidence rate of porcine cysticercosis. |
| [31] | 2014 | Iringa Rural | Estimation of perceived financial cost associated withh porcine cysticercosis | Cross-sectional | 366 respondents | Cost of illness | Estimated annual monetary loss due to porcine cysticercosis of USD 144,449. The estimated monetary burden due to epilepsy management in hospitals and/or by traditional healers was USD 78,592 per annum. |
| [32] | 2014 | Kongwa | Determination of co-endemicity of porcine cysticercosis with other pig endoparasites | Cross-sectional | 309 pigs | Prevalence | Prevalence of 14.9% (95% CI: 10.9-18.9); porcine cysticercosis, 3.9% (95% CI: 1.6-6.1) *Ascaris suum*, 3.2% (95% CI: 1.1-5.2) *Trichuris suis*, 26.3% (95% CI: 21.2-31.5) strongyle species and 11.6% (95% CI: 7.8-15.3) coccidia oocysts. Porcine cysticercosis was more prevalent in wards practising free-range pig rearing (P = 0.000) while GIT helminths were more prevalent in wards practising intensive rearing (P = 0.000). |
| [33] | 2017 | Kongwa | To determine concentrations of cortisol and dehydroepiandrosterone (DHEA) in naturally infected and non-infected pigs | Case-Control (SUA) | 13 infected and 15 non-infected pigs | Difference between means | After the pigs were kept under the same conditions, fed and watered ad libitum, no significant differences were observed between the groups, but a drop in DHEA concentrations was observed in all the pigs. Weight however had an effect on cortisol levels as lean animals had significantly higher cortisol concentrations in both groups, compared to normal pigs. |
| [34] | 2015 | Tanzania | Estimation of societal cost of *T. solium* infections in Tanzania | Systematic Review | 36 scientific articles | DALYs; Cost of illness | For the year 2012 the number of DALYs per thousand person-years for NCC-associated epilepsy was 0.7 (95% UI, 0.2–1.6). Around 5 million USD (95% UI, 797,535–16,933,477)were spentdue to NCC-associatedepilepsy andnearly 3million USD (95% UI, 1,095, 960–5,366,038) were potentially lost due to porcine cysticercosis. |
| [35] | 2017 | SUA experiment | Description of effect of neurocysticercosis on social and feeding behaviours as well as pattern of activities in naturally infected pigs | Case-Control (SUA) | 13 infected and 15 non-infected pigs | Difference between proportions | Sows with NCC spent signiﬁcantly less time at the feeding trough, especially during the second half of the feeding period. Infected sows were also more passive e.g. lying and standing still signiﬁcantly more during a whole day period and showed social isolation compared to non-infected control sows by performing behaviours more distant to their nearest neighbour |
| [36] | 2016 | Tanzania | Modeling of potential control strategies for *T. Solium* | Modelling | Simulation Iterations | Developed model | The model was developed in R and available as an R package (http://cran.r-project.org/package=cystiSim). cystiSim was adapted to an observed setting using field data from Tanzania, but adaptable to other settings if necessary |
| [37] | 2016 | Mbulu | Estimation of prevalence of *T. solium* and associated risk factors in HV+ and HV- individuals | Retrospective Cohort (hospital based) | 170 HIV+ and 170 HIV- controls humans | Difference between proportions | No significant differences between HIV+ and HIV– individuals regarding the sero-prevalence of taeniosis-Ab (0.6% vs 1.2%), CC-Ab (2.4% vs 2.4%) and CC-Ag (0.6% vs 0.0%). TSOL was not associated with CD4 + cell counts, HAART duration or HIV stage. |
| [38] | 2016 | SUA experiment | Description of clinical manifestation associated with neurocysticercosis in naturally infected pigs | Case-Control (SUA) | 16 infected and 15 non-infected pigs | Clinical signs | Some of the observed autonomic signs during a seizure were chewing motions with foamy salivation and ear stiffening. Motor signs included tonic muscle contractions followed by a sudden diminution in all muscle function leading to collapse of the animal. Stereotypic walking in circles was observed on several occasions. At dissection, both pigs had a high number of brain cysticerci (241 and 247 cysticerci). The two pigs with seizures were also older (36 months) compared to the others (18.3 months,  ± 8.2 standard deviation) |
| [39] | 2015 | Mbozi; Mbeya Rural | Identification of risk factors associated with porcine cysticercosis | Case-Control | 43 case & 50 cntrol pig farmers | Odds ratio | Based on logistic regression porcine cysticercosis could be associated with absence or a completely open latrine (p=0.035, OR 5.98, CI: 1.33-43.02) compared to an enclosed latrine. Feeding potato peels to pigs was also associated with increased risk of infection (p=0.007, OR 3.45, CI: 1.43-8.79). |
| [40] | 2015 | Mbeya Rural | Estimation of prevalence of *T. Hydatigena* and *T. Solum* in slaughter pigs | Cross-sectional | 243 pig carcasses | Prevalence | T. hydatigena cysts were found in 6.6 % of the pigs; 80% of cysts were found on the omentum and the rest on the liver, all on the visceral surface. Two pigs were also found infected with *Taenia solium* |
| [41] | 2015 | Mbulu; Mbozi; Kongwa | Comparison of *T. solium* cyst yield between two different preparation mentodhs | Basic research | 23 naturally infected pigs | Cyst fluid yield | This study clearly shows that T. solium cyst preparation in African settings by simple field methods constitutes an effective way to obtain high quality material as source for diagnostic tools and research purposes. |
| [42] | 2014 | Kongwa | Description of anatomical locations of cysts in the CNS and the corresponding inflammation in pig brain | Basic research | 17 naturally infected pigs | Cyst anatomical location | Cysts were distributed in all cerebral lobes (39.7% in the frontal lobe, 20.3% parietal lobe, 20.0% occipital lobe, 19.7% temporal lobe, and 0.4% cerebellum). No cysts were found in the spinal cord. Cysts were localized as follows: 47.9% in the dorsal subarachnoid, 46.9% parenchyma, 4.4% subarachnoid base and 0.9% in the ventricles. |
| [43] | 2014 | Ifakara | Assessment of relationship between exposure to multiple parasitic infections and active convulsive epilepsy | Case-Control (hospital based) | 278 prevalent case and 345 age-matched control humans | Odds ratio | Seropositivity to Onchocerca volvulus, Toxocara canis,Toxoplasma gondii and higher antibody levels (top tertile) to Toxocara canis were associated with an increased prevalence of ACE. Their combined effect on the prevalence of ACE, as determined by the relative excess risk due to interaction was more than additive. The prevalence of *T. solium* antibodies was low (2.8% of cases and 2.2% of controls) and was not associated with ACE in the study area |
| [44] | 2010 | Mbulu | Mapping spatial distribution of porcine cysticercosis in an endemic area | Cross-sectional | Pig cysticercsis data from 21 control villages | Spatial pattern | The K functions revealed a significant overall clustering of porcine cysticercosis incidence for all distances between 600 m and 5 km from a randomly chosen case household based on Ag-ELISA. Lingual examination revealed clustering from 650 m to 6 km and between 7.5 and 10 km. The prevalence study did not reveal any significant clustering by this method. |
| [45] | 2015 | Mbeya Town | Assessment of effect of a computer-based health education tool (The Vicious Worm) on professions' knowledge | Quasi-Experiment | 79 respondents | Change from baseline prevalence | The study subject’s knowledge was significantly improved both immediately after (p=0.001) and two weeks after (p<0.001) the health education. The focus group discussions showed positive attitudes towards the program and the study subjects found ‘The Vicious Worm’ efficient, simple, and appealing. |
| [46] | 2012 | Morogoro | Estimation of prevalence of porcine cysticercosis and transmission risk factors | Cross-sectional | 260 pigs | Prevalence | Four pigs (1.54%:95%CI=0.04–3.1) were found positive by the Ag-ELISA |
| [47] | 2009 | Mbulu | Description of the PRECEDE-PROCEED model in the implementation and evaluation of a health education intervention | Cluster-randomised controlled trial | 393 pigs and pig pig famer respondents | Change from baseline | Process evaluation revealed a good  strategy delivery and a moderate participation of the smallholder pig farmers in the training. The impact evaluation revealed a signiﬁ cant reduction of consumption of pork infected with cysticercosis by the intervention (reduction by 20 per cent, P = 0.005). The outcome evaluation revealed a reduction of the incidence rate of porcine cysticercosis by 43 per cent by the intervention |
| [48] | 2011 | Mbulu | Comparison of two different antibody tests for cysticercosis in humans with end without epilepsy | Cross-sectional | 212 humans with epilepsy followed for 28.6 months | Difference between proportions | The number of NCC lesions and active NCC lesions were significantly associated with a positive antibody result. The electroimmunotransfer blot, developed by the CDC was more sensitive than a commercial western blot, especially in PWE and cerebral calcifications |
| [49] | 2007 | Mbulu | Description of the use of PRECEDE-PROCEED model in the planning of a health education intervtnion for *T. solium* | PRECEDE model planning of intervention | 800 respondents | Developed intervention strategy | Using the PRECEDE model, the authors were able to identify the foci for intervention based on the principles of importance and changeability. |
| [50] | 2011 | Kilolo | Assessment off pig-farmers' knowlege on *T. solium infections* | Cross-sectional | 80 respondents; 3 focus group discussions | Prevalence | Substantial proportion of respondents were aware that porcine cysticercosis (75%), human tapeworm infection (31.2%) and epilespy (20%) could be linked to T. solium. Approximately, 32.5% didn’t have proper knowledge on life cycle of T. solium. About 15% of the surveyed households had no latrines and nearly two- third of pig keepers practiced free range or semi- indoor pig rearing system. About 69% of pig farmers slaughter their pigs at home in which meat inspection is rarely done. |
| [51] | 2015 | Mbulu | Exploration of pig-brain consuption habits | Cross-sectional | 74 respondents | Prevalence | About 29.7% (95% CI: 18.9, 42.4; n = 64) of pork consumers admitted that they  consume pig brains. Nevertheless, 40.6% (95% CI: 28.5, 53.6; n = 64) of the households indicated  that pig brains were consumed. While frying was the preferred method of  cooking pork, boiling in water was the preferred method of cooking the brain. |
| [52] | 2016 | Mbulu | Estimation of performance of lingual exam in the diagnosis of porcine cysticercosis | Secondary analysis of data | Various pig-cysticercosis studies and sizes | Sensitivity; Specificity; Predictive values; Likelihood ratios | Sensitivity of lingual examination in detecting positive results of antigen enzyme-linked immunosorbent assay (Ag-ELISA) was 31.3% in 251 pigs aged 4-7 months and 24.1% in 440 pigs aged 9-12 months. In both age groups the specificity was 99%. The likelihood ratio for a positive lingual examination result was 31.3 and 24.1 in pigs aged 4-7 and 9-12 months, respectively. The likelihood ratio for a negative lingual examination result was 0.7 and 0.8, respectively. Lingual examination of 2-3 months old pigs in three different studies had predictive values for a negative test of 94.9% (95% CI: 93.2, 96.7; n = 609), 84.1% (95% CI: 77.2, 91.0; n = 107) and 100% (95% CI: 100, 100; n = 96). |
| [53] | 2013 | Iringa Rural | Estimation of prevalence of porcine porcine cysticercosis and transmission risk factors | Cross-sectional | 308 pigs; 110 respondents | Prevalence | Prevalence of porcine cysticercosis by lingual exam 23 (7.5%, p<0.001). The prevalence rates was higher in male pigs than female 16 (69.5%) and 7 (30.4%), respectively. Prevalence was higher in villages away from the main roads where there were pigs that customarily ran loose or fed human feaces. The triceps muscle had the highest number of cysts 44 (51.1%) and the diaphragm had the lowest 6 (6.9%). Of 4020 people who sought for treatment in five wards per month, 155 (21.8%) had history of intestinal worms, 6 (2.2%) consumed uninspected pork from peoples house, 82 (74.5%) lack tap water, 13 (12%) had no toilets and 40 (22.7%) practiced free range pig husbandry or tethering method. |
| [54] | 2015 | Hai | Assessment of contribution of *T. solum* to epilepsy | Case-Control | 218 humans with epilepsy; 174 controls | Prevalence | Prevalence of definitive NCC among investigated PWE of 1.1%  (95% CI 0.3–4.0) based on serology combined with CT scan.  Six of 218 PWE had antibodies to T. solium (2.8%; 95% CI 0.6–4.9), compared to none of 174 controls (Fisher’s exact test, P = 0.04). |
| [55] | 2015 | Mbulu | Assessmento of effectiveness of health education on school-children's knowledge and attitudes related to *T. solium* infections | Cluster-randomised controlled trial | Children in 60 schools (30 primary; 30 sec) | Overall score | The overall score (percentage of correct answers) improved by about 10% in all schools after 6 months. Monitoring alone was associated with improvement in scores by about 6%. The intervention was linked to improvements in the attitudeof condemning infected meat but it reduced the attitude of contacting a veterinarian if a pig was found to be infected with cysticercosis |
| [56] | 2015 | Mbozi; Mbeya Rural | Estimation of prevalence of porcine porcine cysticercosis | Cross-sectional | 142 pigs; 90 respondents | Prevalence | Prevalence of porcine cysticercosis by Ag-ELISA was 26% |
| [57] | 2014 | Mbozi | Description of seasonal variations in *T. solium* antigen sero-prevalence in an endemic area | Repeated cross-sectional | In each survey, between 800-1000 pigs tested | Difference between proportions | The first survey revealed a cysticercosis sero-prevalence of 15% (n = 822, 95% CI: 13-18%). The sero-prevalence had significantly increased to 24% (p < 0.001, χ2-test, n = 812, 95% CI: 21-27%) at the time of the 6 month follow-up. At 14-months the sero-prevalence had dropped to 20% (p = 0.053, χ2-test, n = 998, 95% CI: 18-23%) |
| [58] | 1995 | Mbulu; Arusha; Moshi | Determination of the presence of *T. solium* cysticercosis in slaughter pigs and investigate the possible presence of Taiwan Taenia | Cross-sectional | 83 pig carcasses | Prevalence | Overall prevalence of porcine cysticercosis by meat inspection 13.3%. No Taiwan Taenia detected |
| [59] | 2002 | Mbulu | Determination of distribution and density of cysticerci of *T. solium* in distinct carcass sites of naturally infected finishing pigs | Cross-sectional | 24 naturally infected finished pigs | Relative density | The cysticerci in the following order of relative density: psoas muscles (10.5), internal masseter (8.1), external masseter (7.1), triceps brachii (4.9), forelimb (4.0), head muscles (3.8), tongue (3.4), hind limb (3.2), diaphragm (2.4), heart (1.9), abdominal muscles (1.3), trunk muscles (1.1), brain (1.0) and oesophagus (0.3). |
| [60] | 2017 | Mbozi; Mbeya Rural | Assessment of the effect of mass drug administration with praziquantel administered to school-aged children combined with ‘track and treat’ of taeniosis cases in the general popula-tion on the copro-antigen prevalence of taeniasis. | Repeated cross-sectional studies | In total 9064 people were tested (approx 3000 in each of the 3 cross-sectional surveys) | Seroprevalence (coAg-ELISA) | Significant drop in coAg-ELISA seroprevalence post-intervention in both districts (P<0.05) |
| [61] | 1995 | Mbulu; Arusha; Moshi | To explore presence of porcine cysticercosis in pigs slaughtered in Arusha and Kilimanjaro major slaughter slabs, particularly those pigs originated from Mbulu, which was suspected to have porcine cysticercosis | Slaughter-slab record examination and prospective monitoring | Slaughter pigs:  13,514 (in 1985)  13,280 (in 1986)  13,646 (in 1987)  For Mbulu alone:  492 (1985), 840 (1987), | Prevalence | Respective prevalence were:  0.04%, 0.11%, 0.13%, 0.41%, 0.24% based on routine meat inspection. |
| [62] | 2018 | Arusha, Dar es Salaam, Mbeya | To establish the status of porcine cysticercosis in pigs slaughtered in Arusha, Dar es Salaam and Mbeya cities; and to assess the existence of possible risk factors for its transmission in Dar es Salaam city. | Slaughter-slab record examination + questionnaire survey | Slaughter pigs:  (957 in Arusha, 766 in Dar es Salaam and 35418 in Mbeya) | Prevalence | Respective prevalence were:  1.74%, 6.3%, and 0.27% based on routine meat inspection. All infected pigs originated from rural areas.. Potential TSTC transmission risk factors , included lack of centralized pig slaughtering facilities, inadequate meat inspectors, lack/inadequate meat inspection and control, poor knowledge among pork sellers. |
