# Supplementary material for: Taenia solium taeniosis and cysticercosis literature in Tanzania provides research evidence justification for control: A systematic scoping review
Source: PLoS One. 2019 Jun 5;14(6):e0217420. doi: 10.1371/journal.pone.0217420 (PMC6550401; doi:10.1371/journal.pone.0217420)
Supplement: S2 Table — (DOC) [file pone.0217420.s003.doc]

| **Search date** | **Limits** | **Database / other sources** | **Search strategy** |
| --- | --- | --- | --- |
| Feb 07, 2019 | Up to 31/12/2018 | PubMed | Using Medical Subject Headings (MeSH) terms for *Taenia solium* and Tanzania.  Taenia solium (MeSH) + Entry terms:   - Tapeworm, Pork - Pork Tapeworm - Pork Tapeworms - Tapeworms, Pork   Tanzania (MeSH) + Entry terms:   - United Republic of Tanzania - Zanzibar - Tanganyika   **Search**   1. "Taenia solium"[Mesh] (936 items) 2. Pork tapeworm (2247 items) 3. Pork tapeworms (2233 items) 4. Tapeworm, Pork (2247 items) 5. Tapeworms, Pork (2233 items) 6. "Tanzania"[Mesh] (10214 items) 7. United Republic of Tanzania (15676 items) 8. Zanzibar (15672 items) 9. Tanganyika (15834 items) 10. 1 OR 2 OR 3 OR 4 OR 5 (2253 items) 11. 6 OR 7 OR 8 OR 9 (15930 items) 12. 10 AND 11 (45 items)   That is:  (((((("Taenia solium"[Mesh]) OR Pork tapeworm) OR Pork tapeworms) OR Tapeworm, Pork) OR Tapeworms, Pork)) AND (((("Tanzania"[Mesh]) OR United Republic of Tanzania) OR Zanzibar) OR Tanganyika) Filters: Publication date to 2018/12/31. |
| Feb 08, 2019 | Up to 31/12/2018 | African Journals Online (AJOL) | ((((((Taenia solium OR Pork tapeworm) OR Pork tapeworms) OR Tapeworm, Pork) OR Tapeworms, Pork)) AND ((((Tanzania) OR United Republic of Tanzania) OR Zanzibar) OR Tanganyika) |
| Feb 08, 2019 | Up to 31/12/2018 | Google Scholar and Google in general | We searched for specific authors frequently involved in *Taenia solium* for possible more publications that could have been missed from the previous search strategies. In addition, we used these engines to search relevant articles from reference lists of articles retrieved during our primary searches above. |
